# PReS-FINAL-2154: The early predictors of fatal outcome of macrophage activation syndrome in pediatric rheumatic diseases

**DOI:** 10.1186/1546-0096-11-S2-P166

**Published:** 2013-12-05

**Authors:** MM Kostik, NN Abramova, LS Snegireva, LI Vasykina, MF Dubko, VV Masalova, TL Kornishina, TS Likhacheva, IA Chikova, NI Glebova, NV Buchinskaya, OV Kalashnikova, VG Chasnyk

**Affiliations:** 1 Hospital Pediatry, Saint-Petersburg, Russian Federation; 2 ICU, Saint-Petersburg State Pediatric Medical University, Saint-Petersburg, Russian Federation; 3 Immunology, Federal Institute of Public Health «The Nikiforov's Russian Center of Emergency and Radiation Medicine», Saint-Petersburg, Russian Federation

## Introduction

Macrophage activation syndrome (MAS) - is a severe life-threatening hematological condition, complicated different rheumatic diseases. MAS is characterized by uncontrolled proliferation of T cells and macrophages, associated with decreased natural killer (NK) cells and cytotoxic T cell function due to mutations in perforin gene.

## Objectives

The aim of our study was to evaluate early predictors associated with fatal outcomes in pediatric rheumatic disease. These clinical and biomarkers can be useful in the measurement of severity MAS.

## Methods

We have performed retrospective study. Medical charts of children with definite MAS who were admitted to our rheumatology department in 2005-2013 were reviewed. We utilized the A.Ravelli criteria (2002) for detecting MAS. We used the main characteristic clinical and laboratorial markers of MAS only at the moment of MAS confirmation (± 3 days). The patients who developed MAS in the terminal study (irreversible polyorganic damage) were excluded. We evaluated demographic data, data related to MAS. Also we calculated cutoff points for fatal outcomes (ROC-analysis), performed analysis of sensitivity and specificity and identified predictors with Log-Rank analysis and Kaplan-Meier survival curves.

## Results

23 patients (9 boys and 14 girls) with SLE (n = 2), AAV (n = 2), SJIA (n = 17) and virus-associated HLH (n = 2) were included. Median age of MAS was 7.4 years (range:1.5 mo-16.8 y), median time between disease onset and MAS onset 11,4 mo (range:0.4-93.6 mo) and median duration of MAS episode 68 days (range:11-336 days). Fatal outcome was in 4 patients (2 SLE, 1 AAV, 1SJIA).

The main predictors of fatal outcomes were: onset of disease>10.15 years (OR = 42.4, p = 0.004) ongoing glucocorticosteroid treatment at the time of MAS onset (OR = 63.0, p = 0.008), flare or active course of background rheumatic disease (p = 0.015), low sodium Na≤132 mmol/l (OR = 93.0, p = 0.001), no splenomegaly (p = 0.023), no lymphadenopathy (OR = 16.0, p = 0.04), hemorrhagic syndrome (OR = 31.0, p = 0.015), central nervous system involvement (p = 0.015), WBC < 2400 cells in μl (OR = 54.0, p = 0.0001), Prothrombin < 43% (OR = 29.0, p = 0.002) and ESR >56 mm/h (OR = 39.0, p = 0.0007).

## Conclusion

We detected several clinical and laboratorial markers which reflect the severity of MAS. Presence of these signs at onset of MAS can early differentiate the prognostic unfavorable group which required more intensive care.

## Disclosure of interest

None declared.

